# Screening for Anti-Inflammation Quality Markers of Lianhua Qingwen Capsule Based on Network Pharmacology, UPLC, and Biological Activity

**DOI:** 10.3389/fphar.2021.648439

**Published:** 2021-06-11

**Authors:** Yongfeng Zhou, Ming Niu, Dingkun Zhang, Zhenxing Liu, Qinghua Wu, Jiang Chen, Haizhu Zhang, Ping Zhang, Jin Pei

**Affiliations:** ^1^ College of Pharmacy, Chengdu University of Traditional Chinese Medicine, Chengdu, China; ^2^ The Fifth Medical Centre, Chinese PLA People’s Liberation Army General Hospital, Beijing, China; ^3^ College of Pharmacy, Dali University, Dali, China

**Keywords:** Lianhua Qingwen capsule, quality marker, biological activity, network pharmacology, UPLC

## Abstract

Influenza is a common respiratory infectious disease. In China, Lianhua Qingwen capsule (LHQWC), a drug with significant clinical efficacy and few side effects, is commonly used to treat influenza. However, the composition of LHQWC is complicated, and currently used quality control methods cannot ensure its consistency. In this study, combined with its clinical efficacy, the targets of LHQWC were screened using network pharmacology. Then, anti-inflammation quality markers of LHQWC were screened and judged by combined chemical with biological evaluation. Cyclooxygenase-2 (COX-2) was identified as one of the main targets of the anti-inflammatory activity of LHQWC. The rate of inhibition of COX-2 by different batches of LHQWC was determined. Furthermore, seven components of LHQWC were identified. The potential quality markers were screened by spectral-effect relationship. As a result, chlorogenic acid, isochlorogenic acid B, and isochlorogenic acid C were identified and confirmed as anti-inflammatory quality markers of LHQWC. We hope that these findings provide a scientific basis for the accurate quality control of LHQWC and serve as a reference for the quality control of other drugs.

## Introduction

Influenza is an infectious, acute respiratory disease caused by the influenza virus (Guo et al., 2020; Jané et al., 2019). The influenza virus, primarily transmitted by droplets, is highly infectious and was the first infectious disease to be monitored globally (Wang et al., 2012; Wang, 2018). As a result of advancements in medicine and social development, medical and health services have significantly improved. However, the control of influenza remains a challenge. There are 3–5 million cases of severe influenza worldwide every year, among which 290,000–650,000 patients die from flu-related respiratory diseases. Influenza epidemics have seriously threatened the safety of people across the world (Harper et al., 2009; Iuliano et al., 2018; Grohskopf et al., 2020).

Influenza virus can induce upper respiratory tract infection. It has been reported that infected epithelial cells and macrophages produce various bioactive cytokines and chemokines, including chemokines (RANTES, MCP-1, MCP-3, MIP-α and IP-10) and pro-inflammatory cytokines (IL-1β, IL-6, IL-18, TNF-α) (I Julkunen et al., 2001). Therefore, inhibiting the expression of inflammatory factors is one of the main ways to control the occurrence of cytokine storms and reduce the death of influenza patients (Wang et al., 2017; Dai et al., 2018; Gu et al., 2019).

The Lianhua Qingwen capsule (LHQWC), originated from a classical prescription, comprises 13 kinds of traditional Chinese medicine (TCM), including Lianqiao (*Forsythia suspensa* Thunb.Vahl, 255 g), Jinyinhua (*Lonicera japonica* Thunb., 255 g), Gancao (*Glycyrrhiza uralensis* Fisch., 85 g), Zhimahuang (*Ephedra sinica* Stapf, 85 g), Mianmaguanzhong (*Dryopteris crassirhizoma* Nakai, 255 g), Banlangen(*Isatis indigotica* Fort, 255 g), Shigao [(CaSO4• 2H_2_O)255 g], Guanghuoxiang (*Pogostemon cablin* BlancoBenth, 85 g), Chaokuxingren (*Primus armeniaca* L. 85 g), Hongjingtian (*Rhodiola crenulata* (Hook. f. et Thoms.)H. Ohba, 85 g), Yuxingcao (*Houttuynia cordata* Thunb., 255 g), Dahuang (*Rheum palmatum* L. 51 g), l-MENTHOL(*Mentha haplocalyx* Briq. 7.5 g). LHQWC has antibacterial (Wang et al., 2015), anti-inflammatory (Zhang et al., 2014; Xu et al., 2015; Tang et al., 2015), antiviral (Ding et al., 2016; Ding et al., 2017), and antipyretic effects (Niu et al., 2017). It is widely used in the treatment of respiratory diseases caused by various influenza viruses. Anti-inflammation is one of its main antiviral effects and LHQWC can effectively alleviate inflammation and prevent the spread of influenza. It has played a critical role in containing outbreaks caused by respiratory tract viral infections. Because of its significant contributions, LHQWC has been selected as the drug of choice to control influenza by the consensus of state departments and health experts (Yang et al., 2005). LHQWC has also played an important role in the coronavirus disease 2019 (COVID-19) pandemic. A prospective multicenter, open randomized, controlled trial of patients with confirmed COVID-19 that was conducted to test the efficacy of LHQWC showed that LHQWC can improve the patient recovery rate by shortening the recovery time (Hu et al., 2020).

The stability and quality of a drug are essential for ensuring its clinical efficacy. LHQWC, a widely used Chinese patent medicine, is composed of complex chemical components and involves many extraction processes, including volatile oil extraction, alcohol extraction, and water extraction for the volatile, alcohol-soluble, and water-soluble components, respectively (Wang et al., 2015). However, the current method used to evaluate the quality of LHQWC can only measure a single component each time, thereby significantly limiting the manufacturer’s ability to guarantee consistent drug quality and stability.

Prof. Liu Chang-xiao first proposed the concept of quality markers (Q-markers) and their application to accurately control the quality of traditional Chinese medicine and Chinese patent medicine (Liu et al., 2016; Yang et al., 2017). A Q-marker is a chemical substance that indicates the quality of a traditional Chinese medicine or Chinese patent medicine. Q-markers are formed during the production or processing of medicine; they are closely related to the functional properties of various traditional Chinese medicines. (Sun et al., 2020; Xiong et al., 2021). Simultaneously, they can be used to perform qualitative identification and quantitative determination of other traditional Chinese medicines and Chinese patent medicines. Based on this concept, we hope to identify Q-markers correlated with the clinical efficacy of LHQWC and use them for quality control.

In this study, network pharmacology was used to screen the biological targets of LHQWC during treatment for inflammation, and cyclooxygenase-2 (COX-2) inhibitor tests were conducted. A rapid and accurate analytical method using ultra performance liquid chromatography (UPLC) was developed to identify seven chemical components of LHQWC. Overall, 40 batches of LHQWC were evaluated. Furthermore, spectrum-effect relationships were used to screen the Q-markers of LHQWC. Finally, the markers identified in the screening were confirmed with COX-2 inhibition tests.

## Materials and Method

### Instruments

The UPLC system consisted of a Photo-Diode Array (PDA) detector, column compartment, autosampler manager, and a binary solvent delivery pump, connected to Waters Empower two software. Micropipette (Eppendorf, Germany), Ultrapure distilled water was prepared using a Millipore Milli-Q-Plus system (Millipore, Bedford, MA, United States). Screening kits for COX-2 inhibitors were purchased from Beyotiome Biotechnology (Shanghai, China). The fluorescence values were measured by Fluorescence microplate reader (GEMINIXS, United States) and the SOFTmaxPRO software was the production of Molecular Devices Company in United States, Ultrasound (Nanjing, China).

### Chemicals, Reagents, and Materials

Forty batches of LHQWC were collected from YILING Pharmaceutical. Chlorogenic Acid (ChA, No. 151217), Caffeic Acid (CA, No. 151103), Forsythoside A (Fth A, No. 16082602), Isochlorogenic Acid B (3,4-dicaffeoylquinic acid, ICha B, No. 15060708), Rutin (Rt, No. 151023), Isochlorogenic Acid C (4,5-dicaffeoylquinic acid, ICha C, No. 151028) and Phillyrin (Phr, No. 15102801) were acquired from Chengdu Chroma-Biotechnology Co., Ltd. (Chengdu, China), The purity of all standards is more than 98%. Phosphoric acid (HPLC grade) was purchased from Beijing Chemical Works (Beijing, China). Acetonitrile (HPLC grade) was obtained from Fisher Scientific Co., (Fair Lawn, NJ, United States). Celecoxib (Aladdin, No. #E1528116), DMSO (Biovision, United States, No. 2F280547).

### Methods

#### Database Construction

The chemical structures of the composite compounds in LHQWC were obtained from TCMSP https://old.tcmsp-e.com/tcmsp.phpand TCM Database@Taiwan (TDT) (http://tcm.cmu.edu.tw/). Known compound targets were collected from TCMSP and Pharmmapper Database (http://www.lilab-ecust.cn/pharm mapper/, with score ≥ 4) to establish the target data of LHQWC. Gene and protein targets associated with inflammation therapy were collected from the Online Mendelian Inheritance in Man (OMIM) database. Other interacting human proteins of the aforementioned targets were obtained from Database of Interacting Proteins (DIP), and different ID types of the proteins were converted to UniProt IDs.

#### Network Construction and Analysis

In order to elucidate the relationship between chemical components and inflammatory targets, we carried out network pharmacological analysis. The chemical components of LHQWC, the target corresponding to the components, the target corresponding to the inflammation and the target corresponding to the interaction protein were connected into a “chemical components target disease” network file through PPI (http://www.genome.jp/kegg/). The above network was visualized and analyzed by using the software of Cytoscape 2.8.3. Through analyzing the three topological parameters of degree, betweenness centrality and closeness centrality of each node, the possible effective anti-inflammatory target of LHQWC.

#### Chromatographic Conditions

Chromatographic analysis was performed on a Waters Acquity Ultra Performance Liquid Chromatography (UPLC) system equipped with an ACQUITY HSS T3C18 column (2.1 × 100 mm, 1.7 μm) (Water, Milford, MA, United States). The sample was filtered through a syringe filter (0.22 μm) and transferred into the sampling vial. An aliquot of 2.0 μL of the sample solution was injected onto the column maintained at 30°C. The mobile phase consisted of solvent A (methanol) and solvent B (0.1% phosphoric acid in water). A linear gradient was applied at the following conditions: 0–20 min, 10%–28% B; 20–25 min, 28%–48% B; 25–40 min, 48%–60% B; 40–45 min, 60%–10% B. The flow rate was 0.2 ml/min. The effluent was monitored at 330 nm. The method validation was including precision, stability, repeatability, and recovery and the result was shows in Supplementary Table S1.

#### Preparation of Standard Solutions

The mixed standard containing 60 μg/ml ChA, 75 μg/ml CA, 68 μg/ml Fth A, 135 μg/ml ICha B, 64 μg/ml Rt, 80 μg/ml ICha C and 53 μg/ml Phr. Bet was prepared stock into a volumetric flask and dissolved with 10 ml methanol. These solutions were stored in dark glass bottles at 4°C and stable for at least 1 week. Working standard solutions were freshly prepared by diluting suitable amounts of the above solutions with methanol before injection.

#### Preparation of Sample Solutions

Break different batch of LHQWC to powder through a 50-mesh sieve. A total of 0.35 g of powder was accurately weighed and extracted with 20 ml of methanol solution (60%) by ultrasonic extraction for 40 min. Extracted solution was cooled, contributed to weight loss during the extraction procedure, and filtered through a 0.22 μm micropore film to yield the subsequent filtrate solution.

### Determination of Biological Potency

#### Preparation of Sample Solutions

An aliquot of 0.35 g of LHQWC was accurately weighed and added to 10 ml of DMSO; the flask was weighed and extracted by ultrasonic extraction for 30 min. The extracted solution was cooled, and the lost weight was supplied. Next, the mixture was centrifuged at 12,000 rpm for 10 min, the supernatant was collected, and high-concentration sample solutions were obtained for each batch.

#### Determination of COX-2 Inhibition Rate

According to the instructions of the COX-2 inhibitor screening kit, the relative fluorescence of each reaction was measured at an excitation wavelength of 560 nm and an emission wavelength of 590 nm. The inhibition rate of each sample was calculated using the following formula:

$$\text{Inhibition rate}\;\;\left(\%\right)\;=\;\frac{\left(x-y\right)}{x}\;\times \;100\%,$$ (1)

Among them, *x*, and *y* represented the fluorescence value of the 100% enzyme activity control group and the sample group, respectively.

### Biopotency Assay

Celecoxib was used as the standard reference material, and the LHQWC reference material (S40) was diluted with four different concentrations at a ratio of 1:0.5. The inhibition rate of COX-2 was measured in triplicate. The original BP of the LHQWC reference material (S40) was considered 1000 U/μg. Samples from different batches were tested. The BP and confidence interval of COX-2 activity inhibition were calculated. The validation of the method, including its precision, stability, and repeatability as well as the results, is shown in Supplementary Table S4.

### Statistical Analysis

SPSS version 22.0 software was used for correlation analysis of the LHQWC chemical contents and BP. Metaboanalyst (http://www.metaboanalyst.ca/) was used for cluster analysis (parameter: distance measure, pearson *r*, view mode: overview). A *t*-test was performed for significance analysis.

## Results

### Target Screening of LHQWC

Based on the database construction method, a total of 538 compounds of LHQWC, 737 drug targets, 224 targets for inflammation-related diseases, and 676 interactive proteins were screened.

### Chemical Composition-Target-Disease Interactive Network

**FIGURE 1:**
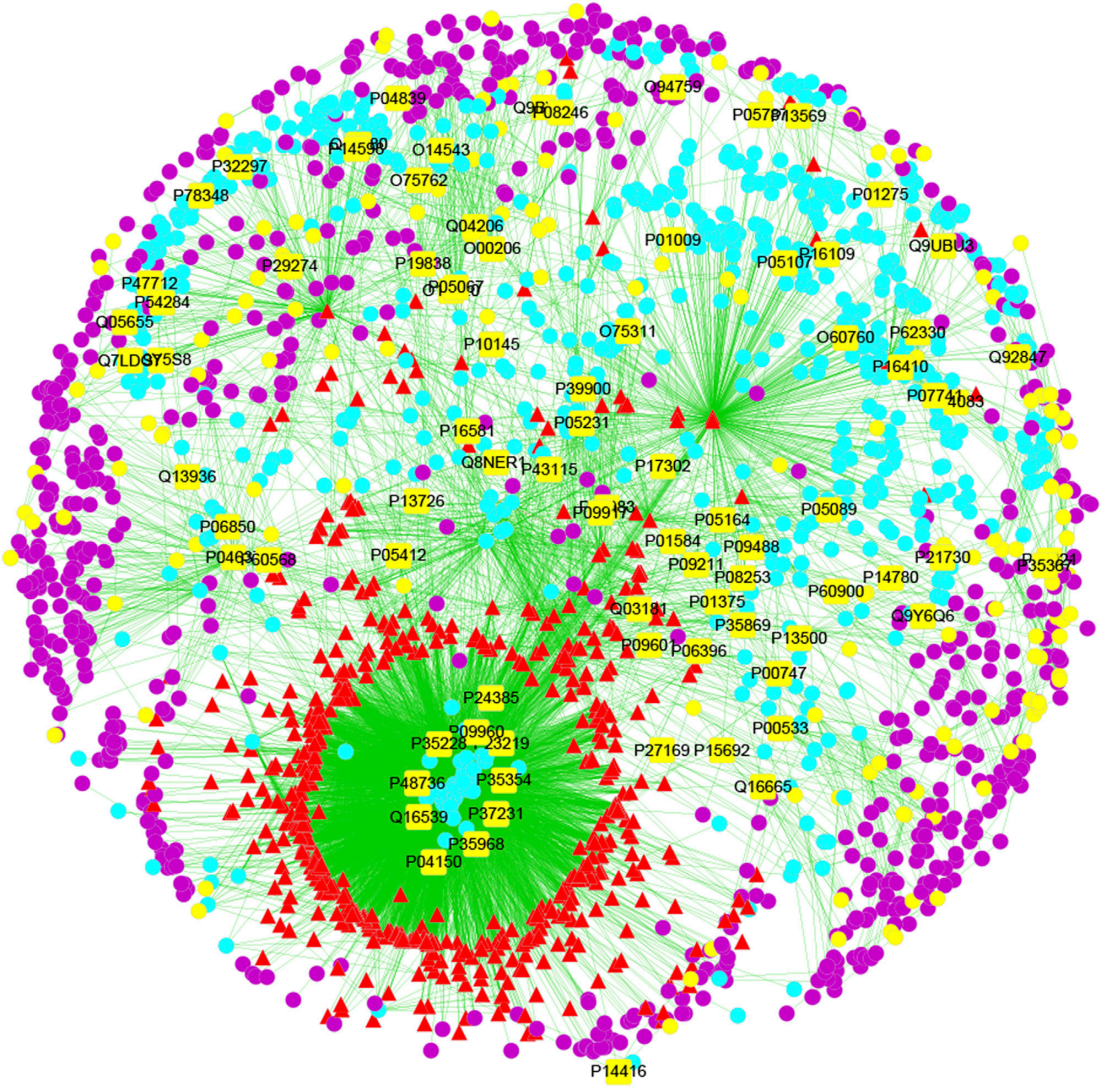
The compound-target-disease interaction network of LHQWC for treating inflammation. The yellow square represents the joint target of drugs and diseases, which is also the most important direct target protein in treating inflammation by LHQWC. Yellow dots represent inflammatory targets. The red triangle represents the predicted possible active ingredients in LHQWC. The blue dot represents the target of LHQWC. The purple dots represent the interaction between the target proteins and the active components of LHQWC.

Network pharmacology can be used to predict the chemical composition–target–disease interaction network of LHQWC for treating inflammation through relevant software; thus, the network relationship between compounds and disease targets can be more clearly and intuitively determined (Figure 1).

### Network and Pathway Analysis

The topological parameters of disease nodes in the network, such as degree, betweenness centrality, and closeness centrality, were analyzed. The median value of degree, betweenness centrality, and closeness centrality was 3, 0.001, and 0.217, respectively. We identified a hub node (above 2-fold of the median degree values) as the candidate target of LHQWC on inflammation if its betweenness centrality and closeness centrality values were more than the corresponding median values. (Table 1 and Supplementary Table S2).

**TABLE 1:** The related topological parameters of LHQW in the treatment of inflammation.

Uniprot No.	Protein names	Betweenness Centrality	Closeness Centrality	Degree
P35354	Cyclooxygenase 2 (COX-2)	0.04735827	0.33775	295
P35228	Nitric oxide synthase	0.05508777	0.338516	219
P23219	Prostaglandin G/H synthase 1	0.01926787	0.324031	193
P37231	Peroxisome proliferator-activated receptor gamma	0.01003448	0.289073	172
Q16539	Mitogen-activated protein kinase 14	0.00309064	0.281557	129
P04637	Cellular tumor antigen p53	0.05583485	0.301631	95
P04150	Glucocorticoid receptor	0.00843658	0.279973	44
P48736	Phosphatidylinositol 4,5-bisphosphate 3-kinase catalytic subunit gamma isoform	0.00107218	0.27249	42
P35968	Vascular endothelial growth factor receptor 2	0.00522803	0.262299	36
P05412	Transcription factor AP-1	0.01471268	0.294283	32
P00533	Epidermal growth factor receptor	0.01367018	0.271994	31
Q04206	Transcription factor p65	0.00964602	0.244616	31
P19838	Nuclear factor NF-kappa-B p105 subunit	0.01004651	0.243363	29
P01375	Tumor necrosis factor	0.02549983	0.307127	20
O14920	Inhibitor of nuclear factor kappa-B kinase subunit beta	0.02520382	0.29194	20
Q16665	Hypoxia-inducible factor 1-alpha	0.00778811	0.23883	18
P24385	G1/S-specific cyclin-D1	0.01198924	0.301849	17
P05067	Amyloid-beta precursor protein	0.01148875	0.277115	17
P15692	Vascular endothelial growth factor A	0.00707075	0.270725	17
Q13936	Voltage-dependent L-type calcium channel subunit alpha-1C	0.00553084	0.256536	13
P16581	E-selectin	0.00670321	0.264223	11
P05231	Interleukin-6	0.0054628	0.269469	9
P35869	Aryl hydrocarbon receptor	0.00459264	0.284586	9
P09917	Arachidonate 5-lipoxygenase	0.00411233	0.302899	9
P09211	Glutathione S-transferase P	0.00382318	0.303118	9
P13500	C-C motif chemokine 2	0.04491083	0.265768	8
P06396	Gelsolin	0.00742301	0.272632	8
P09601	Heme oxygenase 1	0.00195635	0.29734	8
P01584	Interleukin-1 beta	0.00915971	0.269399	7
Q9Y6K9	NF-kappa-B essential modulator	0.01104024	0.257707	23
Q9Y4K3	TNF receptor-associated factor 6	0.0138861	0.243561	22
O15111	Inhibitor of nuclear factor kappa-B kinase subunit alpha	0.00431017	0.245853	19
P25963	NF-kappa-B inhibitor alpha	0.00813931	0.255908	18
P31749	RAC-alpha serine/threonine-protein kinase	0.00782574	0.267914	14
Q99558	Mitogen-activated protein kinase kinase 14	0.00672453	0.241367	14
P09874	Poly [ADP-ribose] polymerase 1	0.00795668	0.24187	12
P25445	Tumor necrosis factor receptor superfamily member 6	0.02383863	0.248425	11
Q99759	Mitogen-activated protein kinase kinase 3	0.0059471	0.218848	10
P19438	Tumor necrosis factor receptor superfamily member 1A	0.00451683	0.2407	10
O15350	Tumor protein p73	0.00197094	0.220185	9
P41182	B-cell lymphoma 6 protein	0.00578785	0.23552	8
Q86WV6	Stimulator of interferon genes protein	0.0029307	0.230176	8
Q13586	Stromal interaction molecule 1	0.001375	0.229418	8
Q13546	Receptor-interacting serine/threonine-protein kinase 1	0.00581301	0.235334	7

### BP Analysis

#### BP Standardization of Reference Substance

Using celecoxib as the control, the COX-2 activity in LHQWC was standardized according to the probit parallel line assay. The BP of celecoxib was measured, and the BP of the reference substance of LHQWC (S40) was defined as 1000 U/μg (Table 2).

**TABLE 2:** Biopotency conversion between LHQW reference and celecoxib.

No.	Reference material (S40)		Celecoxib	
	Doses/μg·mL^−1^	Inhibition/%	Doses/ng·ml^−1^	Inhibition/%
1	15.00	44.54	57.21	47.66
2	30.00	60.48	114.4	59.51
3	60.00	75.12	228.8	71.51
4	120.00	87.74	457.6	89.37

After coordinating the transformation between the inhibition rate and the corresponding concentration of reference substance, BP was calculated using BS2000 software. The result showed that the inhibitory effect of 1 U of S40 was equivalent to that of 3.805 ng of celecoxib.

### BP of COX-2 Activity Inhibition

**FIGURE 2:**
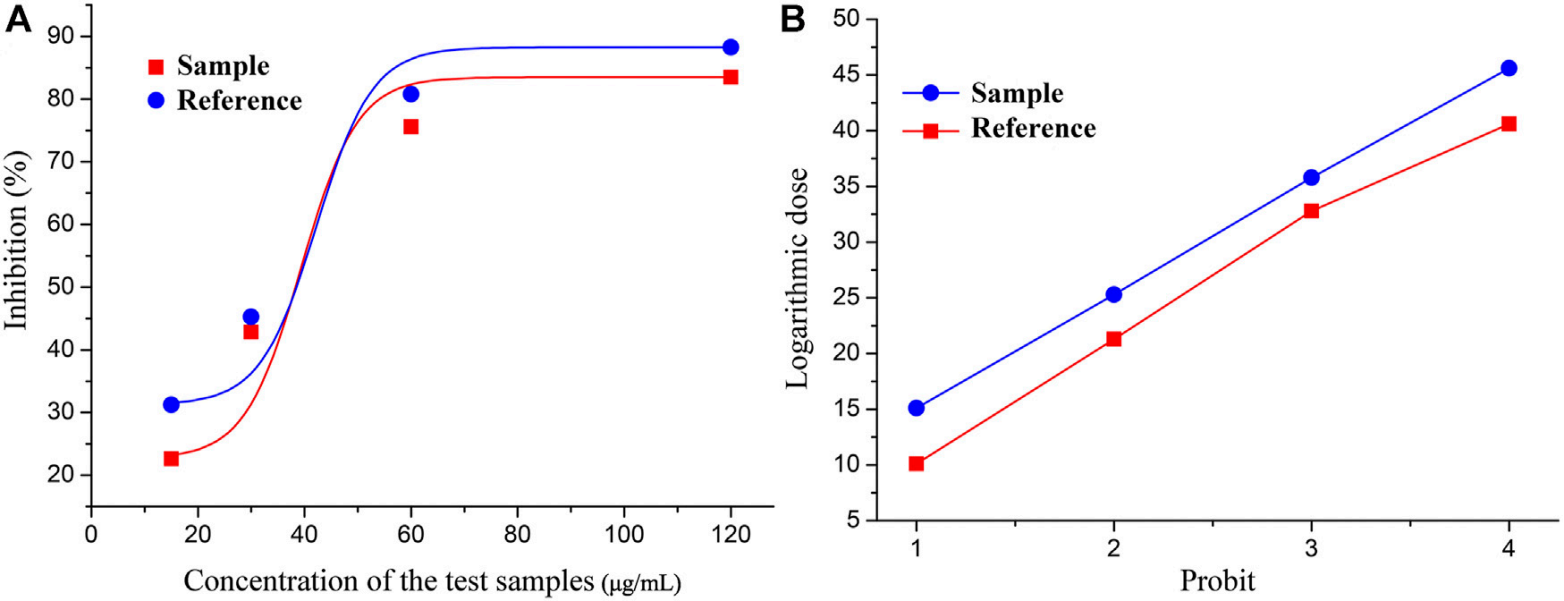
Coordinate transformation of qualitative response: **(A)** The relationship between the concentration of tested drugs and inhibition rate. **(B)** The relationship between logarithmic doses and probit.

The rate of inhibition of COX-2 activity by each batch of LHQWC was calculated. Using S40 as the standard reference, the inhibition rate and the corresponding drug concentration were concurrently transformed. The rate of inhibition of COX-2 activity by each batch of LHQWC was determined using a BP calculation software (Table 3 and Figure 2). The results showed that the inhibitory activity of different batches of LHQWC was 510–1413 U/μg, and their average biological activity was 856 U/μg. Some differences in the rate of inhibition of COX-2 activity by different batches of LHQWC were noted. The BP of batch S26 was the highest, whereas that of S05 was the lowest.

**TABLE 3:** The COX-2 inhibition biopotency result of LHQWC.

No.	R	FL%	PT/U·μg^−1^	No.	R	FL%	PT/U·μg^−1^
S01	0.792	0.13	792	S21	0.795	0.11	795
S02	1.171	0.19	1,171	S22	0.834	0.14	834
S03	0.704	0.1	704	S23	0.74	0.09	740
S04	0.812	0.12	812	S24	0.795	0.09	795
S05	0.645	0.07	510	S25	1.311	0.22	1,311
S06	0.571	0.05	571	S26	1.413	0.33	1,413
S07	0.89	0.16	890	S27	0.51	0.05	645
S08	0.987	0.16	987	S28	0.545	0.07	545
S09	0.645	0.09	645	S29	0.808	0.15	808
S10	0.884	0.14	884	S30	0.715	0.11	715
S11	0.681	0.08	681	S31	0.854	0.16	854
S12	0.984	0.17	984	S32	1.306	0.23	1,306
S13	0.534	0.06	534	S33	0.847	0.12	847
S14	1.29	0.22	1,290	S34	1.072	0.17	1,072
S15	0.67	0.08	670	S35	1.06	0.16	1,060
S16	0.631	0.08	631	S36	0.986	0.25	986
S17	0.552	0.06	552	S37	0.608	0.06	608
S18	1.061	0.14	1,061	S38	0.701	0.07	701
S19	0.817	0.12	817	S39	0.903	0.15	903
S20	1.124	0.17	1,124	S40	—	—	1,000

The results showed that the biological activity of LHQWC had a certain fluctuation. The reasons included the stability of the production process and the stability of the quality of the raw material, which also indirectly showed that it was difficult to ensure the consistency of the curative effect of qualified drugs.

### UPLC Results

**FIGURE 3:**
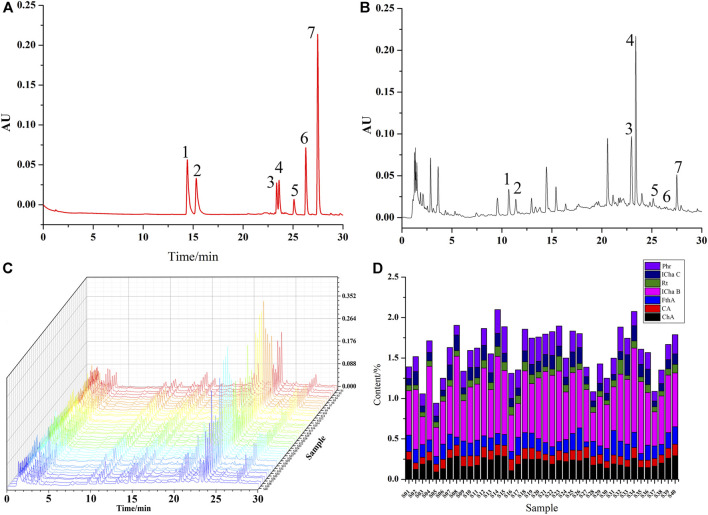
UPLC chromatogram of a mixture of reference substances **(A)** and test samples **(B)**, The fingerprint of LHQWC **(C)**, seven kinds of Seven chemical components in different batches of LHQWC **(D)**. 1. chlorogenic acid, 2. caffeic acid, 3. isochlorogenic acid B, 4. forsythoside A, 5. rutin, 6. isochlorogenic acid C, and 7. phillyrin

Seven types of chemical components in different batches of LHQWC were different based on UPLC results. The total percentages of seven chemical components in three samples (S05, S28, and S37) were lower. The amount of ICha B in different batches of LHQWC was consistently higher than other chemical components (Figure 3 and Supplementary Table S3).

### Results of Cluster Analysis

**FIGURE 4:**
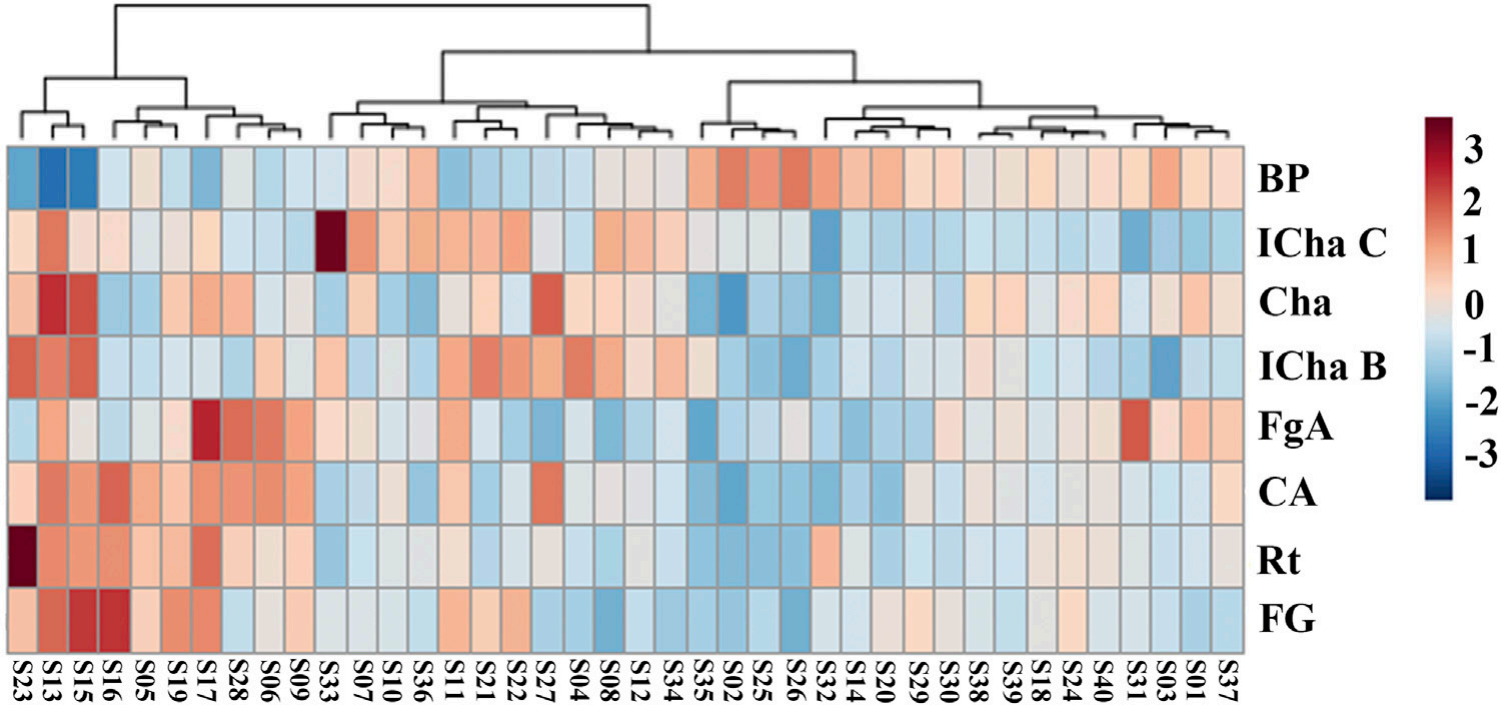
Results of the cluster analysis heat map for 40 batches of LHQWC and seven chemical components. In the heat map, the gradient of deep color to light color indicates high to low intensity of the content of the individual LHQWC compounds.

A heat map providing an intuitive visualization of the data table was constructed. Each colored cell on the map corresponded to a chemical component content value and BP value, with samples in rows and compounds in columns. Cluster analysis results were displayed in a two-dimensional heat map in which the samples were divided into five main clusters (Figure 4). The result shows that the chemical contents and BP are different in the different batches of LHQWC.

### Results of Correlation Analysis of the Spectrum-Effect

**FIGURE 5:**
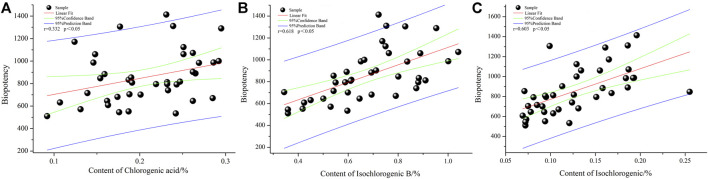
The COX-2 inhibition biopotency and chemical composition content correlation analysis. **(A)**: chlorogenic acid; **(B)**: isochlorogenic acid B and **(C)**: isochlorogenic acid C.

The correlation between the biological potency of COX-2 inhibition and the content of seven chemical components of LHQWC was examined (Table 4 and Figure 5). The chemical content index was used as an independent variable and biological potency as a dependent variable. A total of 40 batches of LHQWC were analyzed. SPSS software was used for multivariate statistical analysis. The results are shown in Table 4 and Figure 5.

**TABLE 4:** Biological activity and chemical composition content correlation analysis.

	ChA		Rt	FgA	FG	ICha C	ICha B	CA
COX-2	r	0.332	0.231	0.269	0.26	0.603	0.618	0.114
	p	*p* < 0.05	*p* > 0.05	*p* > 0.05	*p* > 0.05	*p* < 0.05	*p* < 0.0.05	*p* > 0.05
—	N	40	40	40	40	40	40	40

### Experimental Validation

**FIGURE 6:**
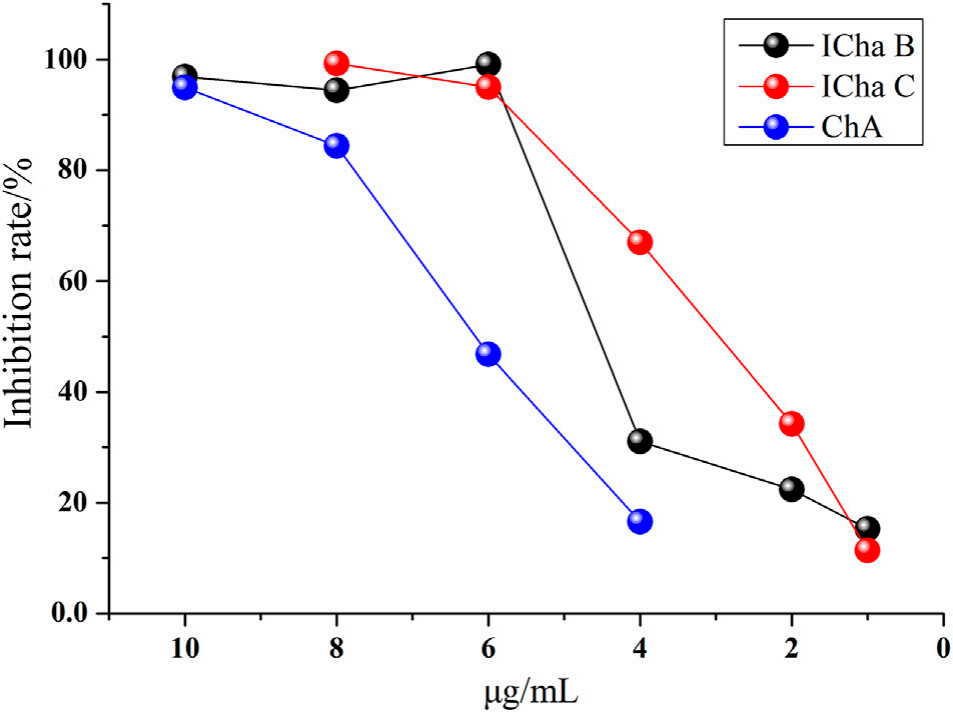
Dose response curves of the COX-2 inhibition rate in the presence of increasing CHA, ICha B, and ICha C concentrations.

The anti-inflammatory activities of ICha B, ICha C, and ChA were validated by determining their inhibition of COX-2 activity at the same concentrations. The result shows that all three compounds inhibited COX-2 activity in a dose-dependent manner; the inhibition rate of ICha B was higher than that of ICha C and ChA (Figure 6). This result is consistent with the abovementioned results.

## Discussion

The quality of a Chinese patent medicine is mainly determined by its active ingredients. However, the chemical composition of Chinese patent medicines varies with complex combinations of active ingredients. Therefore, it is difficult to select an appropriate Q-marker for each medication. In this study, we aimed to identify Q-markers that indicate the active components in a compound as related to efficacy. We used the spectrum-effect correlation method to identify three active components as Q-markers during screening and verify their activity *in vitro*. The results showed that the three chemical components had significant anti-inflammatory activities, thereby providing references for the quality control of LHQWC. However, due to the instrument’s limitations, only seven chemical components were investigated in this experiment; other relevant active components might not have been detected. Further research is needed in this regard.

The chemical composition of Chinese patent medicines is complex because they include many types of medicinal materials. The biological evaluation of these medicines is essential. In this study, based on the COX-2 activity inhibition assay, a titer detection method of anti-inflammatory biological activity was established and optimized. The results show that the method has good reproducibility and stability and can effectively evaluate the anti-inflammatory activity of a drug. Furthermore, this method provides a useful reference for the quality evaluation of other anti-inflammatory traditional Chinese medicines.

*Lonicera japonica* is the JUN in the prescription, and it is the main drug in the treatment of diseases. The three quality markers screened in this study are all from *Lonicera japonica*, which also supports this fact. Network pharmacology analysis found that COX-2 is a target of chlorogenic acid. This study also confirmed the activity of CA from *in vitro* experiments, and the inhibition of COX-2 enzyme activity by CA from *in vivo* experiments have been verified. However, we also found that COX-2 was not the target of ICha B and ICha C in the network pharmacology analysis, but we found that both of them could inhibit the activity of COX-2 enzyme, which indicated that the network pharmacology related components and targets also had certain limitations. The combination of spectrum effect correlation and network pharmacology can better screen active ingredients and find quality markers.

*Forsythia suspensa* is also an important component of LHQWC, its main active ingredients phillyrin and forsythioside A also have certain anti-inflammatory activity (Shi et al., 2011). However, we found that COX-2 may not be the main anti-inflammatory target, and its anti-inflammatory mechanism may be related to NF-κB, JAK-STATS and p38-MAPKs signaling pathway (Pan et al., 2014; Wang et al., 2016).

According to the above research results, we suggest that the quality standard of LHQWC should add chemical components and biological activity. For example, the potency of inhibiting COX-2 enzyme activity should not be less than 500 U/μg based on the reference substance. The average confidence limit was less than 25%. In chemistry, the content of CA should not be less than 0.8 mg/g, ICha B should not be less than 3 mg/g, and ICha C should not be less than 0.7 mg/g.

Although this study preliminarily screened anti-inflammatory quality markers, there are also some shortcomings. In this study, quality markers were screened only from seven components, and some active components may be omitted. Therefore, the analysis of chemical components needs to be further expanded and screened. In addition, LHQWC also have many kinds of activity include antiviral, anti-inflammatory, immunomodulatory except anti-inflammatory. Different activities, the quality markers are also different, so it need to be further studied.

This study described a method to control its quality more accurately by screening for Q-markers based on the spectrum-effect relationship and verifying them. These markers provide a reference for the quality control of LHQWC as well as provide ideas for future research and better service with Chinese medicine worldwide.

## Data Availability

The original contributions presented in the study are included in the article/Supplementary Material, further inquiries can be directed to the corresponding authors.
