# Supplementary material for: Screening for Anti-Inflammation Quality Markers of Lianhua Qingwen Capsule Based on Network Pharmacology, UPLC, and Biological Activity
Source: Front Pharmacol. 2021 Jun 11;12:648439. doi: 10.3389/fphar.2021.648439 (PMC8226139; doi:10.3389/fphar.2021.648439)
Supplement: Supplementary file 1 [file DataSheet1.docx]

Screening for anti-inflammation quality markers of Lianhua Qingwen capsule based on network pharmacology, UPLC, and biological activity

Yongfeng Zhou^1,^^2^, Ming Niu^2^, Dingkun Zhang^1^, Zhenxing Liu^2^, Qinghua Wu^1^, Jiang Chen^1^, Haizhu Zhang^3*^, Ping Zhang^2*^, Jin Pei^1*^

1. College of Pharmacy, Chengdu University of Traditional Chinese Medicine, Chengdu 611137, China

*2.* The Fifth Medical Centre, Chinese PLA People's Liberation Army General Hospital, Beijing 100039, China;

3. College of Pharmacy, Dali University, Dali 671000, China;

***** Corresponding author

Haizhu Zhang: Email, hzningjing@163.com

Ping Zhang: Email, zhp1231@126.com

Jin Pei: Email, peixjin@163.com

|  | Repeatability  RSD/% | Stability  RSD/% | Precision  RSD/% | Linear Relationship | | Recovery  RSD/% |
| --- | --- | --- | --- | --- | --- | --- |
|  |  |  |  | Linear regression R | Linear range（μg） |  |
| ChA | 1.26 | 0.99 | 1.34 | y = 6E+06x-98365 0.9996 | 0.0126~0.2384 | 101.7 |
| CA | 0.87 | 1.24 | 2.05 | y=2E+07x-278272 0.9982 | 0.0241~0.2342 | 103.2 |
| ICha B | 1.35 | 1.09 | 1.46 | y=1E+06x-36052 0.9954 | 0.0102~0.4852 | 102.7 |
| Rt | 3.21 | 1.53 | 0.87 | y=2E+07x-196063 0.9982 | 0.0124~0.3482 | 101.4 |
| ICha C | 1.24 | 2.05 | 2.41 | y=1E+07x-91534 0.9979 | 0.0237~0.3241 | 99.2 |
| FG | 0.48 | 1.76 | 0.74 | y=1E+07x-129526 0.9961 | 0.0109~0.2346 | 98.4 |
| FgA | 0.83 | 0.95 | 1.41 | y=8E+06x-98556 0.995 | 0.0285~0.5342 | 103.2 |

Table S1 The result of method validation about UPLC method

|  | | |
| --- | --- | --- |
| MOL | TCMSP Name | selected Target |
| MOL000098 | quercetin | P00533 P01375 P01584 P04637 P05231 P05412 P09211 P09601 P09917 P13500 P15692 P16581 P23219 P35228 P35354 P35869 P37231 P48736 Q16539 |
| MOL000006 | luteolin | P00533 P01375 P04637 P05067 P05231 P05412 P09211 P09601 P15692 P23219 P35228 P35354 P37231 P48736 Q16539 |
| MOL000422 | kaempferol | P01375 P05412 P09211 P09601 P09917 P16581 P23219 P35228 P35354 P35869 P37231 P48736 Q16539 |
| MOL000173 | wogonin | P01375 P04637 P05231 P05412 P13500 P23219 P35228 P35354 P35968 P37231 P48736 Q16539 |
| MOL001859 | FMT | O14920 P01375 P04150 P09211 P09601 P09917 P23219 P24385 P35228 P35354 |
| MOL000471 | aloe-emodin | P01375 P01584 P04637 P23219 P35228 P35354 P37231 P48736 Q16539 |
| MOL000358 | beta-sitosterol | P04150 P05412 P23219 P35228 P35354 P37231 P48736 Q16539 |
| MOL004480 | acetic acid | O14920 P01375 P09211 P09601 P09917 P23219 P35228 P35354 |
| MOL005916 | irisolidone | P01375 P01584 P05412 P23219 P35228 P35354 P37231 Q16539 |
| rutin | rutin | P01375 P01584 P05231 P09211 P09917 P15692 P35228 Q04206 |
| MOL000513 | 3,4,5-trihydroxybenzoic acid | P04637 P23219 P35228 P35354 P37231 P48736 Q16539 |
| MOL001689 | acacetin | P04637 P23219 P35228 P35354 P37231 P48736 Q16539 |
| MOL001789 | isoliquiritigenin | P16581 P23219 P35228 P35354 P37231 P48736 Q16539 |
| MOL002268 | rhein | P05412 P23219 P35228 P35354 P37231 P48736 Q16539 |
| MOL004328 | naringenin | P09211 P23219 P35228 P35354 P37231 P48736 Q16539 |
| MOL004959 | 1-Methoxyphaseollidin | P23219 P35228 P35354 P35968 P37231 P48736 Q16539 |
| MOL005190 | eriodictyol | P09601 P23219 P35228 P35354 P37231 P48736 Q16539 |
| MOL000354 | isorhamnetin | P23219 P35228 P35354 P37231 P48736 Q16539 |
| MOL000392 | formononetin | P05412 P23219 P35228 P35354 P37231 Q16539 |
| MOL000791 | bicuculline | P23219 P35228 P35354 P35968 P37231 Q16539 |
| MOL001040 | (2R)-5,7-dihydroxy-2-(4-hydroxyphenyl)chroman-4-one | P04150 P23219 P35228 P35354 P37231 Q16539 |
| MOL001484 | Inermine | P23219 P35228 P35354 P37231 P48736 Q16539 |
| MOL001735 | Dinatin | P23219 P35228 P35354 P37231 P48736 Q16539 |
| MOL001736 | (-)-taxifolin | P23219 P35228 P35354 P37231 P48736 Q16539 |
| MOL001763 | 3-(2-hydroxyphenyl)quinazolin-4-one | P23219 P35228 P35354 P37231 P48736 Q16539 |
| MOL001767 | hydroxyindirubin | P23219 P35228 P35354 P37231 P48736 Q16539 |
| MOL001792 | DFV | P23219 P35228 P35354 P37231 P48736 Q16539 |
| MOL001798 | neohesperidin_qt | P23219 P35228 P35354 P37231 P48736 Q16539 |
| MOL001814 | (E)-3-(3,5-dimethoxy-4-hydroxy-benzylidene)-2-indolinone | P23219 P35228 P35354 P37231 P48736 Q16539 |
| MOL001820 | (E)-3-(3,5-dimethoxy-4-hydroxyb-enzylidene)-2-indolinone | P23219 P35228 P35354 P37231 P48736 Q16539 |
| MOL002281 | Toralactone | P23219 P35228 P35354 P37231 P48736 Q16539 |
| MOL002565 | Medicarpin | P23219 P35228 P35354 P37231 P48736 Q16539 |
| MOL002823 | Herbacetin | P23219 P35228 P35354 P37231 P48736 Q16539 |
| MOL002844 | Pinocembrin | P23219 P35228 P35354 P37231 P48736 Q16539 |
| MOL002914 | Eriodyctiol (flavanone) | P23219 P35228 P35354 P37231 P48736 Q16539 |
| MOL003044 | Chryseriol | P23219 P35228 P35354 P37231 P48736 Q16539 |
| MOL003370 | Onjixanthone I | P23219 P35228 P35354 P37231 P48736 Q16539 |
| MOL004576 | taxifolin | P23219 P35228 P35354 P37231 P48736 Q16539 |
| MOL004798 | delphinidin | P23219 P35228 P35354 P37231 P48736 Q16539 |
| MOL004828 | Glepidotin A | P23219 P35228 P35354 P35968 P37231 Q16539 |
| MOL004891 | shinpterocarpin | P23219 P35228 P35354 P37231 P48736 Q16539 |
| MOL004907 | Glyzaglabrin | P23219 P35228 P35354 P37231 P48736 Q16539 |
| MOL004941 | (2R)-7-hydroxy-2-(4-hydroxyphenyl)chroman-4-one | P23219 P35228 P35354 P37231 P48736 Q16539 |
| MOL004966 | 3'-Hydroxy-4'-O-Methylglabridin | P23219 P35228 P35354 P35968 P37231 Q16539 |
| MOL005921 | quercetin 7-O-β-D-glucoside | P23219 P35228 P35354 P37231 P48736 Q16539 |
| MOL000096 | (-)-catechin | P23219 P35228 P35354 P37231 Q16539 |
| MOL000198 | (R)-linalool | P04150 P23219 P35228 P35354 P35968 |
| MOL000239 | Jaranol | P23219 P35228 P35354 P37231 Q16539 |
| MOL000254 | eugenol | P09917 P23219 P35228 P35354 P35869 |
| MOL000417 | Calycosin | P23219 P35228 P35354 P37231 Q16539 |
| MOL000449 | Stigmasterol | P04150 P23219 P35228 P35354 P37231 |
| MOL000492 | (+)-catechin | P23219 P35228 P35354 P37231 Q16539 |
| MOL000497 | licochalcone a | P23219 P35228 P35354 P37231 Q16539 |
| MOL000500 | Vestitol | P23219 P35228 P35354 P37231 Q16539 |
| MOL000522 | arctiin | P35228 P35354 P35968 P37231 Q16539 |
| MOL000676 | DBP | P23219 P35228 P35354 P37231 Q16539 |
| MOL001335 | WLN: Q1R | P23219 P35228 P35354 P37231 Q16539 |
| MOL001733 | EUPATORIN | P23219 P35228 P35354 P37231 Q16539 |
| MOL001793 | (E)-2-[(3-indole)cyanomethylene-]-3-indolinone | P23219 P35228 P35354 P48736 Q16539 |
| MOL001801 | salicylic acid | P09211 P09917 P19838 P23219 P35354 |
| MOL001828 | 3-[(3,5-dimethoxy-4-oxo-1-cyclohexa-2,5-dienylidene)methyl]-2,4-dihydro-1H-pyrrolo[2,1-b]quinazolin-9-one | P23219 P35228 P35354 P37231 Q16539 |
| MOL002235 | EUPATIN | P35228 P35354 P35968 P37231 Q16539 |
| MOL002262 | 5-[(Z)-2-(3-hydroxy-4-methoxy-phenyl)vinyl]resorcinol | P23219 P35228 P35354 P37231 Q16539 |
| MOL002311 | Glycyrol | P35228 P35354 P35968 P37231 Q16539 |
| MOL002610 | ZINC00035529 | P23219 P35228 P35354 P48736 Q16539 |
| MOL002881 | Diosmetin | P23219 P35228 P35354 P37231 Q16539 |
| MOL003095 | 5-hydroxy-7-methoxy-2-(3,4,5-trimethoxyphenyl)chromone | P23219 P35228 P35354 P37231 Q16539 |
| MOL003283 | (2R,3R,4S)-4-(4-hydroxy-3-methoxy-phenyl)-7-methoxy-2,3-dimethylol-tetralin-6-ol | P23219 P35228 P35354 P37231 Q16539 |
| MOL003306 | ACon1_001697 | P23219 P35228 P35354 P37231 Q16539 |
| MOL003358 | Euxanthone | P23219 P35228 P35354 P37231 Q16539 |
| MOL003360 | Norlapachol | P23219 P35228 P35354 P37231 Q16539 |
| MOL003896 | 7-Methoxy-2-methyl isoflavone | P23219 P35228 P35354 P37231 Q16539 |
| MOL004810 | glyasperin F | P23219 P35228 P35354 P37231 Q16539 |
| MOL004815 | (E)-1-(2,4-dihydroxyphenyl)-3-(2,2-dimethylchromen-6-yl)prop-2-en-1-one | P23219 P35228 P35354 P37231 Q16539 |
| MOL004820 | kanzonols W | P23219 P35228 P35354 P37231 Q16539 |
| MOL004824 | (2S)-6-(2,4-dihydroxyphenyl)-2-(2-hydroxypropan-2-yl)-4-methoxy-2,3-dihydrofuro[3,2-g]chromen-7-one | P35228 P35354 P35968 P37231 Q16539 |
| MOL004829 | Glepidotin B | P23219 P35228 P35354 P37231 Q16539 |
| MOL004835 | Glypallichalcone | P23219 P35228 P35354 P37231 Q16539 |
| MOL004836 | echinatin | P23219 P35228 P35354 P37231 Q16539 |
| MOL004838 | 8-(6-hydroxy-2-benzofuranyl)-2,2-dimethyl-5-chromenol | P35228 P35354 P37231 P48736 Q16539 |
| MOL004841 | Licochalcone B | P23219 P35228 P35354 P37231 Q16539 |
| MOL004848 | licochalcone G | P35228 P35354 P35968 P37231 Q16539 |
| MOL004849 | 3-(2,4-dihydroxyphenyl)-8-(1,1-dimethylprop-2-enyl)-7-hydroxy-5-methoxy-coumarin | P35228 P35354 P35968 P37231 Q16539 |
| MOL004883 | Licoisoflavone | P35228 P35354 P35968 P37231 Q16539 |
| MOL004910 | Glabranin | P23219 P35228 P35354 P37231 Q16539 |
| MOL004911 | Glabrene | P23219 P35228 P35354 P37231 Q16539 |
| MOL004912 | Glabrone | P23219 P35228 P35354 P37231 Q16539 |
| MOL004935 | Sigmoidin-B | P35228 P35354 P35968 P37231 Q16539 |
| MOL004945 | (2S)-7-hydroxy-2-(4-hydroxyphenyl)-8-(3-methylbut-2-enyl)chroman-4-one | P23219 P35228 P35354 P37231 Q16539 |
| MOL004957 | HMO | P23219 P35228 P35354 P37231 Q16539 |
| MOL004961 | Quercetin der. | P23219 P35228 P35354 P37231 Q16539 |
| MOL004974 | 3'-Methoxyglabridin | P23219 P35228 P35354 P37231 Q16539 |
| MOL004978 | 2-[(3R)-8,8-dimethyl-3,4-dihydro-2H-pyrano[6,5-f]chromen-3-yl]-5-methoxyphenol | P23219 P35228 P35354 P37231 Q16539 |
| MOL004980 | Inflacoumarin A | P23219 P35228 P35354 P37231 Q16539 |
| MOL004990 | 7,2',4'-trihydroxy－5-methoxy-3－arylcoumarin | P23219 P35228 P35354 P37231 Q16539 |
| MOL004991 | 7-Acetoxy-2-methylisoflavone | P23219 P35228 P35354 P37231 Q16539 |
| MOL005003 | Licoagrocarpin | P23219 P35228 P35354 P37231 Q16539 |
| MOL005007 | Glyasperins M | P23219 P35228 P35354 P35968 P37231 |
| MOL005016 | Odoratin | P23219 P35228 P35354 P37231 Q16539 |
| MOL005017 | Phaseol | P35228 P35354 P35968 P37231 Q16539 |
| MOL005573 | Genkwanin | P23219 P35228 P35354 P37231 Q16539 |
| MOL005842 | Pectolinarigenin | P23219 P35228 P35354 P37231 Q16539 |
| MOL005911 | 5-Hydroxy-7,4'-dimethoxyflavanon | P23219 P35228 P35354 P37231 Q16539 |
| MOL007207 | Machiline | P23219 P35228 P35354 P37231 Q16539 |
| MOL007214 | (+)-Leucocyanidin | P23219 P35228 P35354 P37231 Q16539 |
| MOL000118 | (L)-alpha-Terpineol | P23219 P35228 P35354 P37231 |
| MOL000131 | EIC | P23219 P35228 P35354 P37231 |
| MOL000135 | Deoxyvasicinone | P23219 P35228 P35354 P37231 |
| MOL000348 | 4-[(Z)-3-hydroxyprop-1-enyl]-2,6-dimethoxyphenol | P23219 P35228 P35354 P37231 |
| MOL000432 | linolenic acid | P23219 P35228 P35354 P37231 |
| MOL000675 | oleic acid | P23219 P35228 P35354 P37231 |
| MOL000749 | Linoleic | P23219 P35228 P35354 P37231 |
| MOL000905 | ()-beta-Pinene | P04150 P23219 P35228 P35354 |
| MOL000911 | Terpilene | P04150 P23219 P35228 P35354 |
| MOL000920 | LINALOOL (D) | P23219 P35228 P35354 P37231 |
| MOL001722 | 2-O-beta-D-glucopyranosyl-2H-1,4-benzoxazin-3(4H)-one | P35228 P35354 P37231 Q16539 |
| MOL001739 | zoomaric acid | P23219 P35228 P35354 P37231 |
| MOL001780 | WV | P23219 P35228 P35354 Q16539 |
| MOL001782 | (2Z)-2-(2-oxoindolin-3-ylidene)indolin-3-one | P23219 P35228 P35354 Q16539 |
| MOL001791 | 2,3-dihydro-4-hydroxy-2-oxo-indole-3-acetonitrile | P23219 P35228 P35354 P37231 |
| MOL001803 | Sinensetin | P23219 P35228 P35354 P37231 |
| MOL001805 | 1-methoxyindole-3-carbaldehyde | P23219 P35228 P35354 Q16539 |
| MOL001810 | 6-(3-oxoindolin-2-ylidene)indolo[2,1-b]quinazolin-12-one | P23219 P35354 P35968 Q16539 |
| MOL001818 | Methyl palmitelaidate | P23219 P35228 P35354 P37231 |
| MOL001841 | IES | P23219 P35228 P35354 P37231 |
| MOL002279 | Serotonin | P23219 P35228 P35354 Q16539 |
| MOL002614 | Flavidin | P23219 P35228 P35354 Q16539 |
| MOL002773 | beta-carotene | P05412 P09601 P15692 P35354 |
| MOL003295 | (+)-pinoresinol monomethyl ether | P23219 P35228 P35354 P37231 |
| MOL003308 | (+)-pinoresinol monomethyl ether-4-D-beta-glucoside_qt | P35228 P35354 P37231 Q16539 |
| MOL003310 | 3-ethyl-7hydroxyphthalide | P23219 P35228 P35354 P37231 |
| MOL003656 | Lupiwighteone | P35228 P35354 P37231 Q16539 |
| MOL004805 | (2S)-2-[4-hydroxy-3-(3-methylbut-2-enyl)phenyl]-8,8-dimethyl-2,3-dihydropyrano[2,3-f]chromen-4-one | P35228 P35354 P37231 Q16539 |
| MOL004806 | euchrenone | P35228 P35354 P37231 Q16539 |
| MOL004808 | glyasperin B | P35228 P35354 P35968 P37231 |
| MOL004811 | Glyasperin C | P35228 P35354 P37231 Q16539 |
| MOL004814 | Isotrifoliol | P35228 P35354 P48736 Q16539 |
| MOL004833 | Phaseolinisoflavan | P35228 P35354 P37231 Q16539 |
| MOL004855 | Licoricone | P35228 P35354 P35968 P37231 |
| MOL004857 | Gancaonin B | P35228 P35354 P35968 P37231 |
| MOL004863 | 3-(3,4-dihydroxyphenyl)-5,7-dihydroxy-8-(3-methylbut-2-enyl)chromone | P35228 P35354 P37231 Q16539 |
| MOL004864 | 5,7-dihydroxy-3-(4-methoxyphenyl)-8-(3-methylbut-2-enyl)chromone | P35228 P35354 P37231 Q16539 |
| MOL004879 | Glycyrin | P35228 P35354 P35968 P37231 |
| MOL004885 | licoisoflavanone | P23219 P35228 P35354 P37231 |
| MOL004898 | (E)-3-[3,4-dihydroxy-5-(3-methylbut-2-enyl)phenyl]-1-(2,4-dihydroxyphenyl)prop-2-en-1-one | P35228 P35354 P37231 Q16539 |
| MOL004903 | liquiritin | P35228 P35354 P35968 P37231 |
| MOL004904 | licopyranocoumarin | P35228 P35354 P35968 P37231 |
| MOL004908 | Glabridin | P35228 P35354 P37231 Q16539 |
| MOL004915 | Eurycarpin A | P35228 P35354 P37231 Q16539 |
| MOL004989 | 6-prenylated eriodictyol | P35228 P35354 P37231 Q16539 |
| MOL004993 | 8-prenylated eriodictyol | P35228 P35354 P37231 Q16539 |
| MOL005000 | Gancaonin G | P35228 P35354 P37231 Q16539 |
| MOL005012 | Licoagroisoflavone | P35228 P35354 P37231 Q16539 |
| MOL005020 | dehydroglyasperins C | P35228 P35354 P37231 Q16539 |
| MOL005897 | Labroda | P09917 P23219 P35228 P35354 |
| MOL005918 | phenanthrone | P23219 P35228 P35354 Q16539 |
| MOL000023 | Hemo-sol | P04150 P35228 P35354 |
| MOL000119 | ZINC02040970 | P04150 P35228 P35354 |
| MOL000141 | hydroxytyrosol | P23219 P35228 P35354 |
| MOL000199 | Safrol | P23219 P35228 P35354 |
| MOL000244 | ()-Borneol | P23219 P35228 P35354 |
| MOL000414 | Caffeate | P23219 P35228 P35354 |
| MOL000597 | Neryl acetate | P23219 P35228 P35354 |
| MOL000771 | p-coumaric acid | P23219 P35228 P35354 |
| MOL001398 | Methyllinolenate | P23219 P35354 P37231 |
| MOL001494 | Mandenol | P23219 P35354 P37231 |
| MOL001641 | METHYL LINOLEATE | P23219 P35354 P37231 |
| MOL001721 | Isaindigodione | P23219 P35228 P35354 |
| MOL001723 | 2-O-beta-D-glucopyranosyl-2H-1,4-benzoxazin-3(4H)-one_qt | P23219 P35228 P35354 |
| MOL001725 | 2-O-beta-D-glucopyranosyl-4-hydroxy-2H-1,4-benzoxazin-3(4H)-one_qt | P23219 P35228 P35354 |
| MOL001731 | Dextrin Corn | P23219 P35228 P35354 |
| MOL001732 | IFP | P09211 P23219 P35228 |
| MOL001746 | ELD | P23219 P35354 P37231 |
| MOL001756 | quindoline | P23219 P35354 Q16539 |
| MOL001765 | 2-(1-methoxyindol-3-yl)acetonitrile | P23219 P35228 P35354 |
| MOL001779 | Sinoacutine | P23219 P35228 P35354 |
| MOL001802 | Benzouracil | P23219 P35228 P35354 |
| MOL002203 | Exceparl M-OL | P23219 P35354 P37231 |
| MOL002240 | 5-Carboxy-7-hydroxy-2-methyl-benzopyran-gamma-one | P23219 P35228 P35354 |
| MOL002284 | PIT | P23219 P35354 Q16539 |
| MOL002301 | DLA | P23219 P35228 P35354 |
| MOL002322 | isovitexin | P01375 P35228 P35354 |
| MOL003088 | HYKOP | P23219 P35228 P35354 |
| MOL003290 | (3R,4R)-3,4-bis[(3,4-dimethoxyphenyl)methyl]oxolan-2-one | P35228 P35354 P37231 |
| MOL003312 | suspenolic acid | P23219 P35228 P35354 |
| MOL003322 | FORSYTHINOL | P35228 P35354 P37231 |
| MOL003330 | (-)-Phillygenin | P35228 P35354 P37231 |
| MOL004827 | Semilicoisoflavone B | P35228 P35354 P37231 |
| MOL004856 | Gancaonin A | P35228 P35354 P37231 |
| MOL004866 | 2-(3,4-dihydroxyphenyl)-5,7-dihydroxy-6-(3-methylbut-2-enyl)chromone | P35228 P35354 P37231 |
| MOL004880 | 5,6,7,8-Tetrahydro-2,4-dimethylquinoline | P23219 P35228 P35354 |
| MOL004884 | Licoisoflavone B | P35228 P35354 P37231 |
| MOL004949 | Isolicoflavonol | P35228 P35354 P37231 |
| MOL004964 | (Z)-1-(2,4-dihydroxyphenyl)-3-phenylprop-2-en-1-one | P23219 P35354 Q16539 |
| MOL006218 | Methyl caffeate | P23219 P35228 P35354 |
| MOL006866 | Isosafrole | P23219 P35228 P35354 |
| hexanal | hexanal | P01375 P05412 |
| linalool | linalool | P35228 P35354 |
| MOL000114 | vanillic acid | P23219 P35354 |
| MOL000126 | (-)-nopinene | P23219 P35354 |
| MOL000193 | (Z)-caryophyllene | P23219 P35354 |
| MOL000207 | Methyleugenol | P23219 P35354 |
| MOL000247 | (Z,Z)-farnesol | P23219 P35354 |
| MOL000266 | beta-Cubebene | P35228 P35354 |
| MOL000268 | (1S,5S)-1-isopropyl-4-methylenebicyclo[3.1.0]hexane | P04150 P35354 |
| MOL000421 | nicotinic acid | P23219 P35354 |
| MOL000485 | TMH | P04150 P35354 |
| MOL000635 | vanillin | P05412 P35354 |
| MOL000666 | hexanal | P01375 P05412 |
| MOL000748 | HMF | P23219 P35354 |
| MOL000878 | Farnesylacetone | P23219 P35354 |
| MOL001099 | p-xylene | P04150 P35228 |
| MOL001168 | (1S,2S)-2-isopropenyl-4-isopropylidene-1-methyl-1-vinylcyclohexane | P23219 P35354 |
| MOL001366 | MNN | P35228 P35354 |
| MOL001442 | phytol | P35228 P35354 |
| MOL001456 | citric acid | P35228 P35354 |
| MOL001495 | Ethyl linolenate | P23219 P37231 |
| MOL001606 | BB_NC-0668 | P23219 P35354 |
| MOL001734 | 3-[[(2R,3R,5R,6S)-3,5-dihydroxy-6-(1H-indol-3-yloxy)-4-oxooxan-2-yl]methoxy]-3-oxopropanoic acid | P35228 P35354 |
| MOL001737 | ICO | P35228 P35354 |
| MOL001742 | ASO | P23219 P35354 |
| MOL001743 | 1-[(2R,3S,4S,5S)-3,4-dihydroxy-5-(hydroxymethyl)oxolan-2-yl]pyrimidine-2,4-dione | P35228 P35354 |
| MOL001778 | Sinapaldehyde | P23219 P35354 |
| MOL001781 | Indigo | P23219 P35354 |
| MOL001783 | 2-(9-((3-methyl-2-oxopent-3-en-1-yl)oxy)-2-oxo-1,2,8,9-tetrahydrofuro[2,3-h]quinolin-8-yl)propan-2-yl acetate | P35228 P35354 |
| MOL001835 | BB_NC-1530 | P23219 P35354 |
| MOL001949 | panaxynol | P23219 P35354 |
| MOL002025 | Tereton | P23219 P35228 |
| MOL002042 | thymol | P23219 P35354 |
| MOL002128 | 1,3,8-p-Menthatriene | P23219 P35354 |
| MOL002193 | Cerulignol | P23219 P35354 |
| MOL002207 | 1(3H)-Isobenzofuranone, 3-butyl-3a,4,5,6-tetrahydro-, cis-(-)- | P35228 P35354 |
| MOL002530 | Isobutyl benzoate | P23219 P35354 |
| MOL002850 | butylated hydroxytoluene | P09601 P35968 |
| MOL003006 | (-)-(3R,8S,9R,9aS,10aS)-9-ethenyl-8-(beta-D-glucopyranosyloxy)-2,3,9,9a,10,10a-hexahydro-5-oxo-5H,8H-pyrano[4,3-d]oxazolo[3,2-a]pyridine-3-carboxylic acid_qt | P35228 P35354 |
| MOL003027 | 9-epi-(E)-caryophyllene | P23219 P35354 |
| MOL003043 | (5Z,9Z)-6,10,14-trimethylpentadeca-5,9,13-trien-2-one | P23219 P35354 |
| MOL003103 | Methyl octadeca-8,11-dienoate | P35228 P37231 |
| MOL003112 | (E,Z)-farnesol | P23219 P35354 |
| MOL003117 | Ioniceracetalides B_qt | P35228 P35354 |
| MOL003120 | Loniceracetalide A_qt | P35228 P35354 |
| MOL003177 | Syringaldehyde | P23219 P35354 |
| MOL003302 | forsythidmethylester_qt | P35228 P35354 |
| MOL003319 | 4-Carboxymethylphenol | P23219 P35354 |
| MOL003366 | Matatabiether | P23219 P35354 |
| MOL003520 | Damascenone | P35228 P35354 |
| MOL003587 | Acoradiene | P23219 P35354 |
| MOL003594 | l-Carvyl acetate | P35228 P35354 |
| MOL004285 | MBP | P23219 P35354 |
| MOL004723 | beta-Terpinene | P23219 P35354 |
| MOL004807 | glucuronic acid | P35228 P35354 |
| MOL004837 | Karenzu DK2 | P23219 P35354 |
| MOL004882 | Licocoumarone | P37231 Q16539 |
| MOL004913 | 1,3-dihydroxy-9-methoxy-6-benzofurano[3,2-c]chromenone | P37231 Q16539 |
| MOL004914 | 1,3-dihydroxy-8,9-dimethoxy-6-benzofurano[3,2-c]chromenone | P37231 Q16539 |
| MOL004924 | (-)-Medicocarpin | P35354 P37231 |
| MOL004948 | Isoglycyrol | P35228 P35354 |
| MOL004983 | 5,6,7,8-Tetrahydro-4-methylquinoline | P23219 P35354 |
| MOL005001 | Gancaonin H | P35354 P35968 |
| MOL005008 | Glycyrrhiza flavonol A | P35228 P35354 |
| MOL005018 | Xambioona | P35228 P35354 |
| MOL005030 | gondoic acid | P23219 P37231 |
| MOL005609 | 76897_FLUKA | P23219 P35228 |
| MOL005840 | PANA | P23219 P35354 |
| MOL005903 | WLN: RSR | P23219 P35354 |
| MOL006594 | Eciphin | P23219 P35354 |
| MOL006637 | Psi-ephedrin | P23219 P35354 |
| shigao | shigao | P06396 Q13936 |
| MOL000019 | D-Camphene | P35354 |
| MOL000057 | DIBP | P04150 |
| MOL000122 | 1,8-cineole | P35354 |
| MOL000171 | Guaiol | P35354 |
| MOL000196 | L-Bornyl acetate | P35354 |
| MOL000202 | Moslene | P35354 |
| MOL000211 | Mairin | P04150 |
| MOL000219 | BOX | P35354 |
| MOL000234 | L-Limonen | P35354 |
| MOL000267 | beta-Citronellol | P35354 |
| MOL000270 | CHEBI:7 | P35354 |
| MOL000271 | l-carvone | P35354 |
| MOL000359 | sitosterol | P04150 |
| MOL000474 | (-)-Epoxycaryophyllene | P35354 |
| MOL000475 | anethole | P05412 |
| MOL000671 | ()-Menthol | P35354 |
| MOL000703 | 2-heptanone | P09917 |
| MOL000775 | EEE | P23219 |
| MOL000776 | OXA | P35228 |
| MOL000923 | ACETIC ACID,BORNYL ESTER | P35354 |
| MOL000953 | CLR | P04150 |
| MOL000974 | cuminal | P04150 |
| MOL000991 | cinnamaldehyde | P35354 |
| MOL001129 | l-Verbenone | P04150 |
| MOL001179 | (&#8722;)-Alloaromadendrene | P35228 |
| MOL001237 | o-Acetyltoluene | P04150 |
| MOL001321 | d-mandelonitrile | P35354 |
| MOL001390 | 49070_FLUKA | P35354 |
| MOL001745 | Methyl vaccenate | P37231 |
| MOL001748 | methyl (E)-octadec-8-enoate | P37231 |
| MOL001751 | glucobrassicin_qt | P35354 |
| MOL001755 | 24-Ethylcholest-4-en-3-one | P04150 |
| MOL001771 | poriferast-5-en-3beta-ol | P04150 |
| MOL001774 | Ineketone | P04150 |
| MOL001821 | Methyl 2-ethylhexyl phthalate | P37231 |
| MOL001822 | 5-(methoxymethyl)-2-furoic acid | P35354 |
| MOL001833 | Glucobrassicin-1-Sulfonate_qt | P35354 |
| MOL002003 | (-)-Caryophyllene oxide | P35354 |
| MOL002185 | 7-oxabicyclo-2.2.1-heptane,1-methyl-4-[1-methylethyl]- | P35354 |
| MOL002211 | 11,14-eicosadienoic acid | P37231 |
| MOL002285 | 1-O-Galloyl-glycerol | P35354 |
| MOL002299 | DMR | P35228 |
| MOL002374 | ()-Neomenthol | P35354 |
| MOL002573 | β-patchoulene | P35354 |
| MOL002786 | Apocynin | P35354 |
| MOL002879 | Diop | P37231 |
| MOL003014 | secologanic dibutylacetal_qt | P35354 |
| MOL003036 | ZINC03978781 | P04150 |
| MOL003069 | quinic acid | P35354 |
| MOL003104 | (Z,E)-farnesol | P35354 |
| MOL003111 | Centauroside_qt | P35228 |
| MOL003281 | 20(S)-dammar-24-ene-3β,20-diol-3-acetate | P04150 |
| MOL003294 | Threitol | P23219 |
| MOL003300 | forsythide_qt | P35354 |
| MOL003315 | 3beta-Acetyl-20,25-epoxydammarane-24alpha-ol | P04150 |
| MOL003410 | Ziziphin_qt | P04150 |
| MOL003546 | Aristolone | P35354 |
| MOL003851 | Isoramanone | P04150 |
| MOL004350 | Ruvoside_qt | P04150 |
| MOL004355 | Spinasterol | P04150 |
| MOL004746 | (E,7S,11R)-3,7,11,15-tetramethylhexadec-2-en-1-ol | P35354 |
| MOL004847 | 2,2-DIMETHYLPENTANE | P23219 |
| MOL004944 | Cyclobutanol, 1-ethyl- | P23219 |
| MOL004967 | 3,3-Dimethylpentane | P23219 |
| MOL004985 | icos-5-enoic acid | P37231 |
| MOL004988 | Kanzonol F | P35354 |
| MOL004996 | gadelaidic acid | P37231 |
| MOL005013 | 18α-hydroxyglycyrrhetic acid | P04150 |
| MOL005021 | Mipax | P35354 |
| MOL005893 | Methanoazulene,2,3,6,7,8,8ahexahydro- 1,4,9,9-tetramethyl-,(1R,3aR,7R,- 8aa)- | P35354 |
| MOL005895 | Tricyclo[4.3.1.12,5]undec-3-en-10-ol, 3-(1-methylethyl)-, (1R,2S,5S,6S,10R)-rel- | P35354 |
| MOL005899 | 53111-25-4 | P35354 |
| MOL005902 | 2,2-Dimethylcyclopentylcyclohexane | P35354 |
| MOL005992 | 2,3-dimethyl-1-pentene | P23219 |
| MOL007330 | MENTHOL | P35354 |
| MOL009189 | Tybraine | P35354 |
| MOL009194 | ()-N-Methylpseudoephedrine | P35354 |
| salidroside | sadroside | Q16665 |

Table S2 The relationship between the effective chemical components of LHQWC and the targets inflammation.

| No. | Chlorogenic Acid  （mg/g） | Rutin（mg/g） | Forsythia ester glycosides A  （mg/g） | Forsythia glycosides  （mg/g） | Isochlorogenic Acid C（mg/g） | Isochlorogenic Acid B（mg/g） | Caffeic Acid（mg/g） |
| --- | --- | --- | --- | --- | --- | --- | --- |
| S01 | 2.43 | 0.69 | 2.04 | 1.45 | 0.81 | 5.50 | 0.99 |
| S02 | 1.24 | 0.57 | 1.68 | 1.86 | 1.65 | 7.37 | 0.80 |
| S03 | 1.88 | 0.58 | 1.57 | 1.50 | 0.75 | 3.44 | 0.85 |
| S04 | 2.32 | 0.67 | 1.47 | 1.45 | 1.03 | 9.10 | 1.09 |
| S05 | 0.92 | 0.67 | 0.96 | 1.59 | 0.72 | 3.59 | 0.96 |
| S06 | 1.31 | 0.63 | 1.88 | 1.49 | 0.73 | 5.30 | 1.17 |
| S07 | 2.65 | 0.75 | 1.92 | 2.16 | 1.86 | 5.94 | 1.02 |
| S08 | 2.86 | 0.68 | 0.98 | 1.20 | 1.93 | 10.01 | 1.38 |
| S09 | 1.62 | 0.80 | 1.87 | 2.06 | 0.78 | 5.01 | 1.24 |
| S10 | 1.59 | 0.84 | 1.58 | 2.11 | 1.58 | 6.93 | 1.31 |
| S11 | 1.74 | 0.76 | 1.94 | 2.38 | 1.31 | 6.94 | 1.16 |
| S12 | 2.69 | 0.97 | 1.38 | 2.08 | 1.84 | 8.37 | 1.32 |
| S13 | 2.42 | 0.87 | 1.55 | 2.35 | 1.21 | 5.97 | 1.15 |
| S14 | 2.95 | 1.22 | 1.39 | 2.95 | 1.61 | 9.53 | 1.34 |
| S15 | 2.85 | 1.05 | 1.35 | 3.28 | 1.09 | 7.91 | 1.34 |
| S16 | 1.07 | 1.01 | 0.97 | 3.12 | 1.03 | 4.50 | 1.41 |
| S17 | 1.87 | 0.97 | 2.22 | 2.23 | 0.94 | 4.18 | 1.12 |
| S18 | 2.51 | 1.13 | 1.93 | 2.69 | 1.34 | 7.63 | 1.34 |
| S19 | 2.47 | 1.12 | 1.84 | 3.24 | 1.26 | 6.13 | 1.40 |
| S20 | 2.51 | 0.76 | 1.54 | 3.01 | 1.29 | 7.50 | 0.99 |
| S21 | 2.32 | 0.58 | 1.42 | 2.52 | 1.51 | 8.81 | 0.80 |
| S22 | 1.87 | 0.77 | 1.09 | 2.94 | 1.68 | 8.83 | 1.08 |
| S23 | 2.33 | 1.84 | 1.11 | 2.49 | 1.24 | 8.73 | 1.22 |
| S24 | 2.19 | 0.88 | 1.64 | 2.37 | 0.95 | 5.88 | 1.09 |
| S25 | 2.39 | 0.69 | 2.05 | 2.58 | 1.86 | 7.53 | 1.23 |
| S26 | 2.31 | 0.75 | 2.77 | 1.72 | 1.96 | 7.22 | 1.28 |
| S27 | 2.62 | 0.67 | 0.62 | 1.18 | 0.93 | 6.46 | 1.39 |
| S28 | 1.76 | 0.68 | 1.85 | 1.14 | 0.72 | 3.59 | 1.09 |
| S29 | 1.91 | 0.68 | 1.08 | 2.41 | 0.94 | 6.10 | 1.15 |
| S30 | 1.39 | 0.54 | 1.57 | 1.87 | 0.85 | 5.42 | 0.86 |
| S31 | 1.90 | 0.81 | 3.03 | 2.01 | 0.71 | 5.42 | 1.10 |
| S32 | 1.77 | 1.79 | 1.87 | 3.04 | 0.99 | 8.29 | 1.08 |
| S33 | 1.54 | 0.49 | 1.94 | 2.05 | 2.55 | 8.01 | 0.87 |
| S34 | 2.62 | 0.89 | 1.89 | 1.77 | 1.87 | 10.41 | 1.31 |
| S35 | 1.48 | 0.58 | 0.82 | 1.87 | 1.55 | 8.89 | 0.89 |
| S36 | 1.46 | 0.96 | 1.86 | 2.06 | 1.92 | 6.51 | 0.91 |
| S37 | 1.63 | 0.62 | 1.53 | 1.21 | 0.69 | 4.24 | 0.96 |
| S38 | 2.01 | 0.61 | 1.30 | 1.69 | 0.91 | 5.99 | 1.01 |
| S39 | 2.62 | 0.77 | 1.92 | 1.89 | 1.13 | 7.12 | 1.23 |
| S40 | 2.92 | 1.06 | 2.16 | 2.38 | 1.29 | 6.65 | 1.42 |

Table S3 Content determination results of seven active ingredient in LHQW capsule

|  | Repeatability  RSD/% | Stability  RSD/% | Precision  RSD/% |
| --- | --- | --- | --- |
|  |  |  |  |
| COX-2 Inhibition | 9.32 | 8.56 | 5.14 |

Table S4 The result of method validation about Biopotency method (n=6)
